# Multilayered epithelium in a rat model and human Barrett's esophagus: Similar expression patterns of transcription factors and differentiation markers

**DOI:** 10.1186/1471-230X-8-1

**Published:** 2008-01-11

**Authors:** Xiaoxin Chen, Rong Qin, Ba Liu, Yan Ma, Yinghao Su, Chung S Yang, Jonathan N Glickman, Robert D Odze, Nicholas J Shaheen

**Affiliations:** 1 Cancer Research Program, Julius L. Chambers Biomedical/Biotechnology Research Institute, North Carolina Central University, 700 George Street, Durham, NC 27707, USA; 2 Susan Lehman Cullman Laboratory for Cancer Research, Department of Chemical Biology, Ernest Mario School of Pharmacy, Rutgers, The State University of New Jersey, Piscataway, NJ 08854, USA; 3 Center of Esophageal Diseases and Swallowing, Division of Gastroenterology and Hepatology, University of North Carolina at Chapel Hill, Chapel Hill, NC 27599, USA; 4 Department of Medicine and Center for Health Services Research, Vanderbilt University Medical Center, Nashville, TN 37232, USA; 5 Department of Pathology, Brigham and Women's Hospital, Harvard Medical School, Boston, MA 02115, USA

## Abstract

**Background:**

In rats, esophagogastroduodenal anastomosis (EGDA) without concomitant chemical carcinogen treatment leads to gastroesophageal reflux disease, multilayered epithelium (MLE, a presumed precursor in intestinal metaplasia), columnar-lined esophagus, dysplasia, and esophageal adenocarcinoma. Previously we have shown that columnar-lined esophagus in EGDA rats resembled human Barrett's esophagus (BE) in its morphology, mucin features and expression of differentiation markers (*Lab. Invest. 2004;84:753–765*). The purpose of this study was to compare the phenotype of rat MLE with human MLE, in order to gain insight into the nature of MLE and its potential role in the development of BE.

**Methods:**

Serial sectioning was performed on tissue samples from 32 EGDA rats and 13 patients with established BE. Tissue sections were immunohistochemically stained for a variety of transcription factors and differentiation markers of esophageal squamous epithelium and intestinal columnar epithelium.

**Results:**

We detected MLE in 56.3% (18/32) of EGDA rats, and in all human samples. As expected, both rat and human squamous epithelium, but not intestinal metaplasia, expressed squamous transcription factors and differentiation markers (p63, Sox2, CK14 and CK4) in all cases. Both rat and human intestinal metaplasia, but not squamous epithelium, expressed intestinal transcription factors and differentiation markers (Cdx2, GATA4, HNF1α, villin and Muc2) in all cases. Rat MLE shared expression patterns of Sox2, CK4, Cdx2, GATA4, villin and Muc2 with human MLE. However, p63 and CK14 were expressed in a higher proportion of rat MLE compared to humans.

**Conclusion:**

These data indicate that rat MLE shares similar properties to human MLE in its expression pattern of these markers, not withstanding small differences, and support the concept that MLE may be a transitional stage in the metaplastic conversion of squamous to columnar epithelium in BE.

## Background

Barrett's esophagus (BE) is characterized by replacement of esophageal squamous epithelium by intestinalized columnar epithelium, most commonly due to chronic gastroesophageal reflux disease. Patients with BE are at increased risk for the development of esophageal adenocarcinoma, which is now the most rapidly increasing type of cancer in Western countries [1].

The pathogenesis of intestinal metaplasia (IM) in BE is poorly understood. For instance, theories regarding the origin of stem cells that give rise to intestinalized columnar epithelium include origin from esophageal squamous epithelium or the submucosal glands [2-5]. However, regardless of the site of origin, it is generally believed that pluripotent stem cells are triggered by inflammatory mediators and/or gastroesophageal refluxate to differentiate into intestinalized columnar epithelium [5,6]. Recent studies have focused on the potential roles of acid and bile, inflammatory mediators, and intestinal transcription factors in the pathogenesis of human BE [6-11]. Despite these efforts, the mechanism underlying the development of IM in BE remains unclear.

Multilayered epithelium (MLE), first described in 1997, is a hybrid epithelium that expresses cytokeratins of both squamous and columnar differentiation [12]. It is composed of multiple layers of cells that appear squamous in the basal portion and columnar in the superficial aspects. Several studies have shown that MLE is predominantly located at, or near, the neo-squamocolumnar junction in patients with BE. Multilayered epithelium has also been shown to correlate with BE and reflux esophagitis. Thus, it has been postulated that MLE may represent a transitional stage in the squamous to columnar transition in BE [13-16].

In our laboratory, we utilized a rat surgical model to study BE. In this model, an esophagoduodenal anastomosis was created to induce gastroesophageal reflux. Sprague-Dawley rats subjected to this procedure developed esophageal adenocarcinoma [17]. Subsequently, we modified this procedure by performing an esophagogastroduodenal anastomosis (EGDA) in order to induce reflux of gastric and duodenal contents into the esophagus. Previous studies have shown that more than 50% of rats developed columnar-lined esophagus and adenocarcinoma 40 weeks after surgery [18]. In rats, MLE frequently also occurs at the neo-squamocolumnar junction, but occasionally in the mid-esophagus as well. Rat columnar-lined esophagus resembles human BE in its morphology, mucin features and in its expression of intestinal differentiation markers, such as keratin 7, keratin 20, Das-1, villin, and trefoil factor 1 [19].

The purpose of this study was to evaluate and compare MLE in our rat model to that in humans with established BE, in order to gain further insight into the role of MLE in the development of BE. To perform this study, serial paraffin-embedded esophageal tissue sections from 32 EGDA rats were evaluated for expression patterns of transcription factors and differentiation markers of esophageal squamous epithelium and intestinal columnar epithelium not previously evaluated. Mucosal biopsy samples containing MLE from 13 human patients with established BE were also studied for comparison with the rat samples.

## Methods

### Tissue samples

Esophagogastroduodenal anastomosis was performed on 32 rats as described previously. [18] In brief, a surgical anastomosis was created between the gastroesophageal junction and the duodenum, on the antimesenteric border, with accurate mucosal to mucosal opposition, in 8-week-old male Sprague-Dawley rats. The animals were treated with iron dextran (4 mg Fe/kg/week, i.p.) for 40 weeks. After the animals were sacrificed by CO2 asphyxiation, the esophagus was removed, fixed in 10% buffered formalin, Swiss-rolled, processed, and embedded in paraffin. Serial sections of tissue blocks from all 32 rats (300 to 500 sections per block) were then made for this study.

For comparison, coded (with all identifiers removed) archival formalin-fixed paraffin-embedded esophageal mucosal biopsies from 13 patients with MLE in BE were obtained from the files of the Department of Pathology, Brigham and Women's Hospital, Boston, MA. The study protocol was approved by the Institutional Review Board. Ten serial sections of each case were used in this study. Tissue sections from these 13 patients were specifically selected because they all contained MLE, as described previously [16].

### Pathological evaluation

In rat tissues, one out of every twenty serial tissue sections was stained with hematoxylin and eosin, and then counterstained with Alcian blue at pH2.5. Tissue sections were evaluated for the presence of BE and MLE [19]. In brief, BE was characterized by the occurrence of IM, which was defined by the presence of goblet cells in the esophagus. Multilayered epithelium consists of 4 to 8 layers of cells that show squamous differentiation in the basal portion and columnar differentiation in the superficial layers. Serial sections that contained MLE were identified and selected for immunohistochemical staining.

Tissue sections of human samples were simultaneously stained with H&E and the presence of BE and MLE was confirmed. The presence or absence of squamous epithelium was also noted.

### Immunohistochemical staining

The following four groups of markers were studied (Table 1): *(1) *squamous transcription factors (p63, Sox2); *(2) *squamous differentiation markers (CK14, CK4); *(3) *intestinal transcription factors (Cdx2, GATA4, HNF1α); and *(4) *intestinal differentiation markers (villin, Muc2).

**Table 1:** Transcription factors and differentiation markers used in this study

Antigen	Description	Expression Pattern	Antibody Source	Catalogue No.	Conc.
p63	Transcription factor essential for squamous epithelium	Nuclei of basal and parabasal squamous cells	Santa Cruz Biotechnology (Santa Cruz, CA)	sc-8431 (mouse mAb)	0.2 μg/ml
Sox2 (SRY-related HMG box gene 2)	Transcription factor essential for upper gastrointestinal epithelium	Nuclei of epithelial cells in oral cavity, esophagus and stomach	Santa Cruz Biotechnology	sc-17320 (goat pAb)	4 μg/ml
CK14 (cytokeratin 14)	Squamous differentiation marker	Cytoplasm of basal squamous cells	Novocastra Laboratories Ltd. (Newcastle upon Tyne, UK)	NCL-LL002 (mouse mAb)	1:40
CK4 (cytokeratin 4)	Squamous differentiation marker	Cytoplasm of parabasal squamous cells	Sigma-Aldrich (St. Louis, MO)	C5176 (mouse mAb)	1:500
Cdx2 (*caudal*-related homeobox 2)	Intestinal transcription factor	Nuclei (and occasionally cytoplasm) of columnar and goblet cells	BioGenex (San Ramon, CA)	MU392-UC (mouse mAb)	1:50
GATA4 (GATA-binding protein 4)	Intestinal transcription factor	Nuclei of columnar and goblet cells	Santa Cruz Biotechnology	sc-1237 (goat pAb)	0.25 μg/ml
HNF1α (hepatocyte nuclear factor 1α)	Intestinal transcription factor	Nuclei of columnar and goblet cells	Santa Cruz Biotechnology	sc-6547 (goat pAb)	0.4 μg/ml
Villin	Intestinal differentiation marker	Cytoplasm of columnar and goblet cells	NeoMarkers (Fremont, CA)	MS-1499 (mouse mAb)	5 μg/ml
Muc2 (mucin 2)	Intestinal differentiation marker	Cytoplasm of goblet cells	Santa Cruz Biotechnology	sc-15334 (rabbit pAb)	1:500

Briefly, paraffin-embedded tissue sections were deparaffinized, rehydrated, and pretreated by heating the slides for 5–10 min in 10 mM citrate buffer. Immunohistochemical staining was performed with the ABC kit (Vector Labs, Capenteria, CA) according to the manufacturer's instructions. The sources of the primary antibodies, catalogue numbers, and working concentrations are listed in Table 1. Normal serum or phosphate buffered saline were used instead of the primary antibodies, as negative controls. Both positive and negative control slides were processed in parallel. Alcian blue staining (1% in 3% acetic acid, pH2.5, for 10 min) was performed after immunohistochemical staining on the same slides to visualize cells that produce acidic mucin.

Immunohistochemical staining for each marker was scored by one pathologist (R.Q.) as either positive or negative in the epithelium of interest. Areas of squamous epithelium, MLE and IM were scored separately. For analysis of p63, Sox2, Cdx2, GATA4, and HNF1α, epithelium with strong nuclear staining was counted as positive. Cytoplasmic staining of Cdx2 was also noted. Cytoplasmic staining was counted as positive for analysis of CK4, CK14, villin and Muc2. The frequency of positive staining for each marker in each epithelial type was then calculated (Table 2).

**Table 2:** Expression of transcription factors and differentiation markers in tissue samples from EGDA rats and humans

Antigen	EGDA Rat Samples No. of positively stained foci/No. of total foci (%)			Human Biopsy Samples No. of positively stained foci/No. of total foci (%)		
		
	Squamous epithelium	MLE	Intestinal metaplasia	Squamous epithelium	MLE	Intestinal metaplasia
p63^a^	128/128 (100%)	85/85 (100%)	0/142 (0%)	11/11 (100%)	5/13 (38.5%)	0/3 (0%)
Sox2	114/114 (100%)	71/71 (100%)	0/128 (0%)	11/11 (100%)	13/13 (100%)	0/3 (0%)
CK14^a^	113/113 (100%)	76/76 (100%)	0/139 (0%)	11/11 (100%)	0/13 (0%)	0/3 (0%)
CK4	116/116 (100%)	30/55 (54.5%)	0/129 (0%)	11/11 (100%)	4/13 (30.8%)	0/3 (0%)

Cdx2	0/114 (0%)	0/77 (0%)	127/127 (100%)	0/11 (0%)	2/13 (15.4%)	3/3 (100%)
GATA4	0/114 (0%)	0/70 (0%)	130/130 (100%)	0/11 (0%)	0/13 (0%)	2/3 (66.7%)
HNF1α	0/100 (0%)	0/54 (0%)	111/111 (100%)	N/A	N/A	N/A
Villin	0/115 (0%)	15/56 (26.8%)	133/133 (100%)	0/11 (0%)	2/13 (15.4%)	3/3 (100%)
Muc2	0/114 (0%)	0/75 (0%)	129/129 (100%)	0/11 (0%)	0/13 (0%)	3/3 (100%)

^a ^Statistically significant difference between rat and human MLE (p < 0.0001).

### Statistical analysis

Statistical analysis was conducted with Fisher exact test for frequency data. For each immunohistochemical stain, the frequency of positive staining between rat and human MLE was compared.

## Results

### Histopathology of rat and human samples

Twenty nine EGDA rats (29/32, 90.6%) contained BE, defined by the presence of IM in the esophagus. Multilayered epithelium was detected in 18 of 32 (56.3%) EGDA rats. The mean number of MLE foci per rat was 2.4 (range 1 to 6). Most MLE foci (91%) were located at the neo-squamocolumnar junction, whereas fewer were located distant from the junction, or in the mid-esophagus. The morphologic features of MLE in the rat model closely resembled that of human patients [14,16,19].

Human samples containing MLE were intentionally selected for this study. In addition, eleven samples also had squamous epithelium within their BE biopsies.

### Expression of squamous transcription factors and differentiation markers in rat and human samples (Figure 1; Table 2)

**Figure 1:**
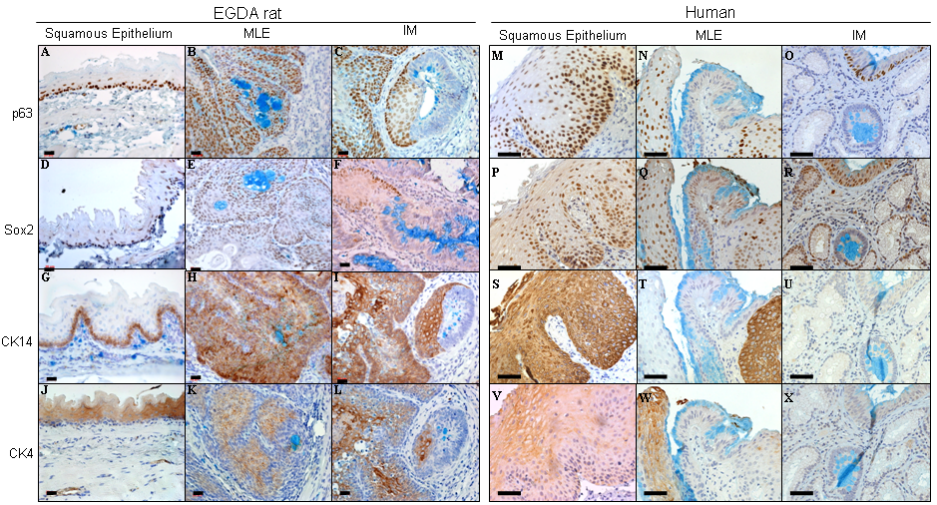
Expression of squamous transcription factors (p63, Sox2) and differentiation markers (CK4, Ck14) in squamous epithelium, MLE and IM of EGDA rats and humans. All the tissue sections were stained immunohistochemically with a specific antibody, and then histochemically with Alcian blue. Light blue: Alcian blue staining of acidic mucin; dark blue: hematoxylin staining for nuclei; dark brown: immunohistochemical staining. Size bar equals 20 μm in rat samples, or 40 μm in human samples.

Both rat and human squamous epithelium uniformly expressed squamous transcription factors (p63 and Sox2) and differentiation markers (CK14 and CK4) in all cases (Figure 1A, D, G, J, M, P, S, V). P63 and Sox2 were both expressed in the nuclei of squamous epithelial cells in the basal cell layer. CK14 was expressed in the cytoplasm of basal cells, and CK4 in suprabasal cells.

Neither rat nor human IM expressed any of these transcription factors or differentiation markers (Figure 1C, F, I, L, O, R, U, X).

Both rat and human MLE expressed squamous transcription factor, Sox2, in the nuclei of basal cell layer of all cases (Figure 1E, Q), similar to squamous epithelium. p63 was expressed in 100% (85/85) of rat MLE (Figure 1B). However, only 38.5% (5/13) of human MLE (Figure 1N) expressed p63 (p < 0.0001 *vs*. rat MLE).

Of the two squamous differentiation markers (CK14 and CK4), CK14 was expressed in basal cells of rat MLE in all cases, similar to squamous epithelium (Figure 1H). However, in human MLE, CK14 was negative in all 13 cases (p < 0.0001, *vs*. rat MLE; Figure 1T). CK4 was expressed in 54.5% (30/55) of rat MLE (Figure 1K), and in 30.8% (4/13) of human MLE (Figure 1W). Such a difference was not statistically significant (p > 0.05).

### Expression of intestinal transcription factors and differentiation markers in rat and human samples (Figure 2; Table 2)

**Figure 2:**
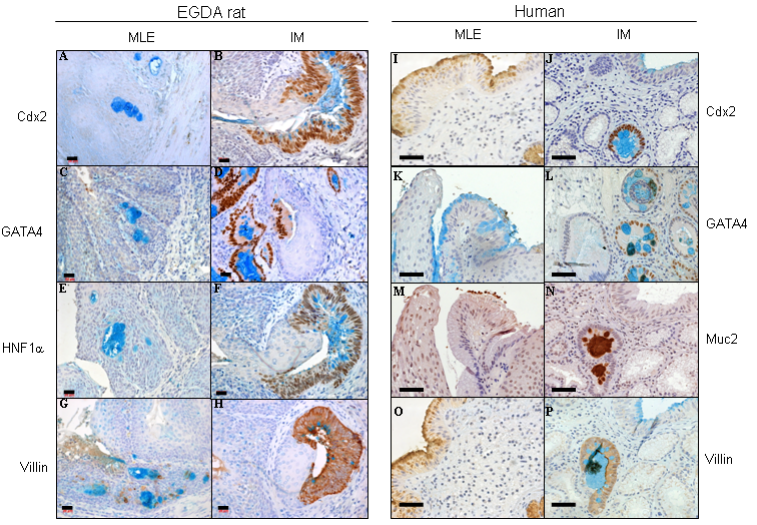
Expression of intestinal transcription factors (Cdx2, GATA4, HNF1α) and differentiation markers (villin, Muc2) in MLE and IM of EGDA rats and humans. All the tissue sections were stained immunohistochemically with a specific antibody, and then histochemically with Alcian blue. Light blue: Alcian blue staining of acidic mucin; dark blue: hematoxylin staining for nuclei; dark brown: immunohistochemical staining. Size bar equals 20 μm in rat samples, or 40 μm in human samples.

Neither rat nor human squamous epithelium expressed any of the intestinal transcription factors (Cdx2, GATA4 and HNF1α) and differentiation markers (villin and Muc2) (figures not shown). However, both rat IM (Figure 2B, D, F, H) and human IM (Figure 2J, L, N, P) expressed all intestinal markers studied, except for one case of human IM in which GATA4 was not expressed.

Rat MLE did not express Cdx2, GATA4, and HNF1α (Figure 2A, C, E) in any of the cases. Similar to rat MLE, human MLE did not express GATA4 and Muc2 (Figure 2K, M). Interestingly although Cdx2 was not expressed in the nuclei in any cases, it was detected in the cytoplasm of two cases (Figure 2I).

Villin, an intestinal differentiation marker, was expressed in 26.8% (15/56) of rat MLE (Figure 2G), and in 15.4% (2/13) of human MLE (Figure 2O). Such a difference was not statistically significant (p > 0.05).

## Discussion

Multilayered epithelium, a hybrid epithelium consisting of squamous cells in the basal portion and columnar cells in the superficial layers, was first described in esophageal biopsies in patients with BE [16]. Human MLE expressed differentiation markers of both squamous and intestinal columnar epithelium, suggesting that it might represent a transitional stage in the metaplastic conversion of squamous to columnar epithelium in BE [12,14]. In this study, we compared the phenotype of rat MLE versus human MLE in order to gain further insight into the nature of MLE, and its potential role in the development of BE.

We focused on squamous transcription factors (p63, Sox2) and differentiation markers (CK4, CK14), and intestinal transcription factors (Cdx2, GTAT4, HNF1α) and differentiation markers (villin, Muc2). Transcription factors represent "master switch" genes, which regulate expression of differentiation-related molecules with both structural and functional specificity, and, thus, mediate metaplasia. Using immunohistochemical staining we found that both rat and human MLE simultaneously expressed markers of both squamous and intestinal differentiation, and that with minor exceptions, rat and human MLE shared similar expression patterns of these markers. For instance, we found that rat MLE is more likely to express p63 and CK14 compared to human MLE.

The squamous transcription factors and differentiation markers evaluated in this study include p63, Sox2, CK4 and CK14. P63 is a critical initiator of epithelial stratification and a key regulator of cell adhesion and survival in progenitor cells in squamous epithelium [20-22]. In mouse esophagus, when basal cells become differentiated, p63 is down-regulated and eventually turned off in terminally differentiated superficial cells [15,23]. In p63-deficient mice, embryonic esophageal epithelium appears columnar containing both ciliated and goblet-like cells [24]. Previous studies have shown that p63 is expressed in the basal and suprabasal cell layers, and in submucosal glands, of human esophageal squamous epithelium [15,25]. p63 in esophageal epithelial cells was downregulated when they were exposed to bile and acid [26]. In this study, p63 was detected in all cases of rat MLE, but in only 38.5% (5/13) of human MLE (p < 0.0001). This result suggests that rat and human MLE show differences in their native esophageal epithelium, and that basal cells of rat MLE maintain a higher degree of "squamous" differentiation compared to rat MLE. It is known that rat esophagus is covered by keratinized squamous epithelium and human esophagus by non-keratinized squamous epithelium.

As a member of the *Sry*-like high mobility group domain protein family, Sox2 may play an important role in the development of the upper gastrointestinal tract. It is expressed in the pharynx, esophagus, and stomach of chicken gut, but not in the lower gastrointestinal tract, where Cdx1 and Cdx2 are present and play an important role in cell differentiation [27,28]. Sox2 mutations are associated with esophageal atresia in anophthalmia-esophageal-genital syndrome [29], and its down-regulation is associated with intestinal metaplasia in the stomach [30,31]. Hypomorphic Sox2 mice developed metaplastic changes of morphology and gene expression in the esophagus [32]. Our data showed that Sox2 was expressed in both rat and human MLE in all cases. In intestinal metaplasia, Sox2 was negative in both rat and human samples. Recently, Sox2 was reported to be silenced by promoter methylation in intestinal-type gastric cancer [33]. Further studies are needed to understand the mechanisms by which Sox2 is turned off in metaplastic columnar epithelium.

CK4 and CK14 are differentiation markers of esophageal squamous epithelium. CK14 is typically expressed in basal cells and CK4 in parabasal cells [34]. Interestingly, in our study, CK14 was not expressed in human MLE, but it was expressed in all cases of rat MLE. This expression pattern was similar to that of p63 in rat and human MLE, likely due to the fact that CK14 is a transcriptional target of p63 [35]. Nevertheless, these data support the theory that rat MLE maintains a higher level of "squamous" differentiation compared to human MLE. In the presence of gastroesophageal reflux, human esophagus was more likely to lose squamous differentiation features.

We also evaluated intestinal transcription factors (Cdx2, GATA4 and HNF1α) and differentiation markers (villin and Muc2). As a member of the *Caudal-*related homeobox gene family, Cdx2 is important for skeletal and intestinal development. Cdx2 plays an important regulatory role in the development of intestinal metaplasia in the foregut, and in cancer development in the colon [36,37]. Cdx2 is normally not expressed in squamous cells of the normal human esophagus. However, one previous study showed that a low level of *Cdx2 *mRNA was detected in biopsy samples of esophageal squamous epithelium in patients with esophagitis [11,38]. In BE, Cdx2 is expressed in goblet and non-goblet cells [39]. In fact, several Cdx2-regulated genes are known "markers" of BE, such as villin, guanylate cyclase C, and sucrase isomaltase [40-42]. We recently demonstrated that treatment of esophageal epithelial cells with acid, bile acids or both resulted in promoter demethylation and activation of *Cdx2 *[43]. In this study, Cdx2 was negative in all rat MLE samples, which suggests that Cdx2 is not expressed at an early stage of columnar metaplasia. However, in human MLE, two cases expressed Cdx2 in the cytoplasm, although none of them showed nuclear expression. Similarly, cytoplasmic expression of Cdx2 has been reported in the columnar epithelium of colon, bile duct and stomach [44-46]. The significance of cytoplasmic accumulation of Cdx2 is still unclear.

GATA4 belongs to a subfamily of the GATA transcription factor family involved in differentiation of mesoderm and endoderm-derived tissues. GATA4, 5 and 6 are expressed in the stomach and small intestine, but not in the esophagus [47-49]. HNF1α is a liver-enriched homeodomain-containing transcription factor, which regulates many genes in liver and pancreas [50,51]. Intestinal transcriptional factors (GATA4, HNF1α, and Cdx2) have been shown to play cooperative roles in regulating the expression of several marker genes in intestinal epithelium and human BE [40,50,51]. In this study, neither rat nor human MLE expressed GATA4 or HNF1α, suggesting that GATA4 and HNF1α are probably not involved at an early stage of conversion from squamous to columnar epithelium in the esophagus.

Villin is an actin-bundling protein and a marker of human BE [19,52-54]. Muc2 is a differentiation marker of intestinal and tracheobronchial goblet cells [55], and is known to be regulated by *Cdx2 *in esophageal epithelial cells [9,43]. In this study, villin was expressed in a small percentage of rat and human MLE, whereas Muc2 was not expressed in either type of tissues. These data suggest that the mucinous cells in MLE do not possess some biological properties of fully developed goblet cells. It is possible that, during the metaplastic conversion of squamous to columnar epithelium, villin is expressed at an early phase, whereas Muc2 is expressed only when phenotypically mature goblet cells develop. Although villin is a possible transcriptional target of Cdx2 [41], our data suggest that other factors may regulate its expression in MLE.

The mechanism of intestinal metaplasia is still unclear. Our data in rat model strongly support the theory that stem cells reside in the esophagus. Three theories have been proposed for the development of intestinal metaplasia in human BE. The *de novo *metaplasia theory proposes that the stem cells of inflamed squamous epithelium are stimulated by reflux to undergo columnar metaplasia. The transitional zone metaplasia theory suggests that epithelial cells at the squamocolumnar junction colonize the gastric cardia or distal esophagus in response to reflux. The esophageal gland duct cell metaplasia theory proposes that stem cells are located in the neck of submucosal gland ducts, and they are stimulated to expand upon deep ulceration of the mucosa and submucosa [5]. However, rats do not have submucosal glands or ducts in their esophagus, and rat MLE is commonly found in the mid-esophagus unrelated to the neo-squamocolumnar junction after EGDA surgery. Thus, development of MLE in EGDA rats supports pluripotent stem cells in the esophagus as the cellular origin of intestinal metaplasia. This hypothesis is consistent with previous studies suggesting gut regenerative cell lineage as the likely cellular origin of BE [56]. Although MLE is likely a precursor lesion of BE, it can not be excluded that MLE may be a distinct multi-directional differentiation type of epithelium, which may not develop into BE.

## Conclusion

In summary, this study shows that rat MLE is similar to human MLE in the expression pattern of markers of squamous and intestinal differentiation. Further studies are warranted to determine how these markers are modulated by gastroesophageal reflux, and how they cooperate with each other to facilitate columnar metaplasia.

## Abbreviations

BE: Barrett's esophagus; Cdx: *caudal*-related homeobox; CK: Cytokeratin; EGDA: Esophagogastroduodenal anastomosis; GATA4: GATA-binding protein 4; HNF1: Hepatocyte nuclear factor 1α; IM: Intestinal metaplasia; MLE: Multilayered epithelium; Muc2: Mucin 2; Sox2: SRY-related HMG box gene 2.

## Competing interests

The author(s) declare that they have no competing interests.

## Authors' contributions

XC designed the study, participated in the data interpretation, and drafted the manuscript. RQ read the slides and performed the statistical analysis. BL and YM carried out the sectioning and immunostaining. YS, CSY, JNG, RDO, and NJS participated in the design of the study and the data interpretation. JNG and RDO also helped to draft the manuscript. All authors read and approved the final manuscript.

## Pre-publication history

The pre-publication history for this paper can be accessed here:


